# Marine Collagen from Alternative and Sustainable Sources: Extraction, Processing and Applications

**DOI:** 10.3390/md18040214

**Published:** 2020-04-15

**Authors:** Daniela Coppola, Maria Oliviero, Giovanni Andrea Vitale, Chiara Lauritano, Isabella D’Ambra, Salvatore Iannace, Donatella de Pascale

**Affiliations:** 1 Department of Marine Biotechnology, Stazione Zoologica Anton Dohrn, Villa Comunale, 80121 Napoli, Italy; daniela.coppola@szn.it (D.C.); chiara.lauritano@szn.it (C.L.); 2 Institute of Biosciences and BioResources (IBBR), National Research Council, Via Pietro Castellino 111, 80131 Naples, Italy; 3 Institute of Polymers, Composites and Biomaterials, National Research Council, P.le E. Fermi 1, Portici, 80055 Naples, Italy; maria.oliviero@cnr.it (M.O.); salvatore.iannace@cnr.it (S.I.); 4 Institute of Biochemistry and Cell Biology (IBBC), National Research Council, Via Pietro Castellino 111, 80131 Naples, Italy; giovanniandrea.vitale@ibbc.cnr.it; 5 Department of Integrative Marine Ecology, Stazione Zoologica Anton Dohrn, Villa Comunale, 80121 Napoli, Italy; isabella.dambra@szn.it

**Keywords:** marine collagen, marine gelatin, sustainable sources, by-catch, discards, protocols

## Abstract

Due to its unique properties, collagen is used in the growing fields of pharmaceutical and biomedical devices, as well as in the fields of nutraceuticals, cosmeceuticals, food and beverages. Collagen also represents a valid resource for bioplastics and biomaterials, to be used in the emerging health sectors. Recently, marine organisms have been considered as promising sources of collagen, because they do not harbor transmissible disease. In particular, fish biomass as well as by-catch organisms, such as undersized fish, jellyfish, sharks, starfish, and sponges, possess a very high collagen content. The use of discarded and underused biomass could contribute to the development of a sustainable process for collagen extraction, with a significantly reduced environmental impact. This addresses the European zero-waste strategy, which supports all three generally accepted goals of sustainability: sustainable economic well-being, environmental protection, and social well-being. A zero-waste strategy would use far fewer new raw materials and send no waste materials to landfills. In this review, we present an overview of the studies carried out on collagen obtained from by-catch organisms and fish wastes. Additionally, we discuss novel technologies based on thermoplastic processes that could be applied, likewise, as marine collagen treatment.

## 1. Introduction

Collagen is a complex macroprotein which groups 20%–30% of all proteins found in living organisms [1], and represents the main structural component of the extracellular matrix in all connective tissues (i.e., skin, bones, ligaments, tendons and cartilage) and interstitial tissues of the parenchymal organs.

Thanks to its unique properties, collagen is well-known as a structural support for biomedical devices, dermal implants and emerging health applications, as well as being largely used in nutricosmetic, food and beverages. Due to its high-water absorption capacity, collagen is a good candidate for texturizing, thickening and gel formation. Moreover, it has interesting properties related to surface behavior, which involves emulsion, foam formation, stabilization, adhesion and cohesion, protective colloid functions and film-forming capacity. Also, collagen is a good surface-active agent, with its ability to penetrate lipid-free interfaces [2].

Collagen can be utilized in a variety of applications because of its biocompatibility and excellent degradability [3,4]. Furthermore, it is known that collagen is a molecule with weak immunogenicity, which decreases the possibilities of rejection when it is ingested or injected into a different body. Although this molecule has already low antigenicity, this property can be enhanced by modifying it to suppress any immune response [5,6]. Additionally, collagen peptides and gelatin (denatured collagen) have been widely utilized in different fields such as food, medicine, cosmetics, leather and film industries, diagnostic imaging, and therapeutic delivery [7].

For many years, most available collagen was extracted from discards from the bovine and porcine processing industries, but during the last few decades the use of collagen from these sources has been limited. The use of porcine and bovine-derived products is occasionally prevented by dietary regimes, due to specific needs or personal choices. It is forbidden by religious constraints, to Muslims, Hindus and Jews who make up 38.4% of the global population [8]. Moreover, the use of bovine-derived products became a concern for a wider section of the population during the bovine spongiform encephalopathy (BSE), transmissible spongiform encephalopathy (TSE) and foot-and-mouth disease (FMD) crises that occurred over the last few decades in all areas of the world, mostly in the United Kingdom and Asia. Bovine-derived products might be a vehicle of transmission of these diseases and therefore their use has been severely limited (regulations CE n. 999/2001 and UE n. 142/2011). Hence, there is an urgent need to find novel alternative sources of collagen.

Recently, marine organisms have received consideration as promising sources of collagen, because they have no limitations in use for any religions and there are no reports of possible transmissible diseases. In particular, biomass derived from the activities of fish-processing industries and fisheries (fish and sea urchin wastes, undersized fish and by-catch organisms such as jellyfish, sharks, starfish, sponges) might become an important, yet underexploited, source of collagen [9,10,11,12,13,14]. The use of discarded and underused biomass will contribute to the development of a sustainable pipeline to obtain collagen with a significantly reduced environmental impact.

In this review, we present a general overview of the studies carried out on by-catch organisms and wastes from fish and sea urchin processing industries in order to isolate collagen. We discuss the structure of collagen, the established methodologies for collagen extraction from these marine sources, and its applications. We also discuss novel technologies based on thermoplastic processes that have been investigated for other types of proteins, and that might be applied to marine collagen.

## 2. Sustainable Marine Sources of Collagen

Collagen is found not only in terrestrial organisms, but also in a variety of marine species. In particular, the skin and bones of fish and sharks, sea urchin waste and by-catch organisms such as jellyfish and starfish, have high collagen content, as shown in Table 1.

Fish waste is very abundant worldwide and several studies, projects, and local and international authorities have focused on how to use this valuable waste [10,12,34,35,36,37,38,39,40]. Its utilization has recently increased in order to enhance the economic value of by-catch and fish by-products for biotechnological applications, and also because of the urgent need to reduce the amount of waste for contemporary societies.

Fish processing industries produce large amounts of fish waste every year that represent approximately 25% of the total production. The waste mainly consists of bones, skin, scales, and fins, which constitute over 70% of fish. Currently, part of these wastes are utilized as feedstuff because they contain almost the same amount of proteins as fish flesh [41]. Nevertheless, the majority of this waste is not fully used and most of it is discarded [42], creating a fundamental problem for the environment. 

Determinations of the protein composition in fish discards have highlighted that skin, bones and scales of fish are rich in collagen, as displayed in Table 1. The yield of collagen extracted from these discards can reach up to higher than 50% in dry mass, even though the number of fish examined is relatively limited compared to the number of fish species used in food industries. Therefore, this waste material has the potential to be exploited as an eco-friendly and low-cost collagen source.

Waste is generated routinely by seafood and sea urchin processing and by fishing activities. The implementation of the landing obligation, as part of the recent reform of the EU Common Fisheries Policy (CFP), requires that catches of regulated commercial species, including undersized animals which cannot be used for direct human consumption, and endangered species (such as several shark species) to be landed and accounted for quota. An estimate of discards throughout the Mediterranean suggests that they constitute around 18.6% of the catches [43], which makes them a compelling ecological and economic problem.

The sea urchin peristomial membrane, a food industry waste, has been suggested as sustainable and eco-friendly source of fibrillar collagen to produce membranes for regenerative medicine applications [24].

In addition to fish waste and sea urchins, jellyfish (pelagic Cnidaria, in particular scyphomedusae) appear to be increasing worldwide [44,45,46] and are often abundantly caught in fish nets. Although jellyfish have been a primary ingredient in traditional Chinese food for many centuries [47], they are considered a nuisance because of their negative interactions with human activities, such as tourism, aquaculture, and fishery [48].

In light of the apparent increase of jellyfish abundance and their occurrence as by-catch in fishing nets, some studies focused on determining their protein content. Analyses indicated that their organic matter is made up mainly by collagen [49], thus they may be considered as a promising source of this fibrous protein.

Scaffolds for tissue engineering made of collagen extracted from the scytphomedusa *Rhizostoma pulmo* were successfully implanted into a mouse model and showed optimal adsorption and biocompatibility properties [50]. These findings suggest that collagen derived from scyphomedusae may become a suitable replacement for bovine-derived collagen.

A recent review has shown that rhizostome scyphomedusae contain more collagen than semaeostomeae scyphomedusae [51], as shown in Table 1. The collagen content in *Rhopilema esculentum* is significantly high, considering that the value in Table 1 is based on dry mass. The other two scyphomedusae, *Stomolophus meleagris* and *Rhopilema asamushi*, are commonly consumed by Eastern populations, and contain the highest percentage of collagen on a wet mass basis of all the scyphomedusae where determination of collagen was made, as shown in Table 1. 

Table 1 displays that native rhizostome scyphomedusae, such as *R. pulmo* and *Cotylorhiza tuberculata* in the Mediterranean Sea also present high collagen content. The collagen extracted from the invasive rhizostome *Catostylus tagi* has already been considered for exploitation [29], suggesting this species as a suitable source of collagen.

## 3. Collagen Structure

**Figure 1:**
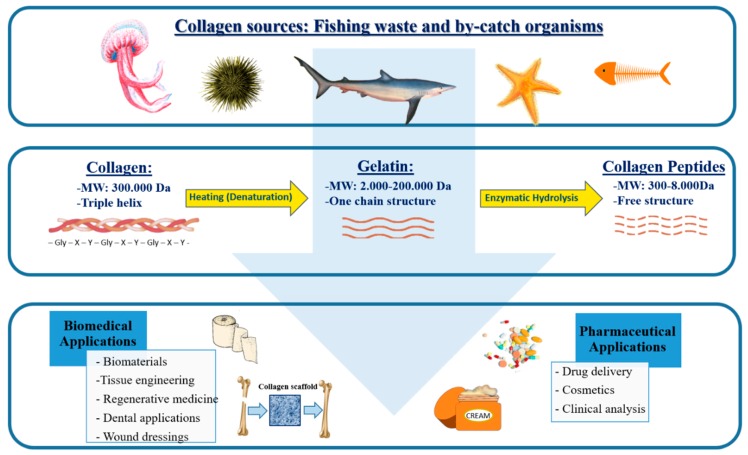
The classical structure and applications of marine collagen, gelatin, and collagen peptides extracts from sustainable marine sources.

There are 28 distinct types of collagen named with Roman numeral designations (I–XXVIII) in chronological order of discovery [3,52]. Collagens are generally made up of three long helicoidally-shaped chains of amino acids (about 1050 in each helix). The basic structure of the chains is the triplet, where glycine bonds with two other amino acids, and with the repeating sequence (Gly-X-Y)_n_; typically, proline or hydroxyproline are often located at position Y. The chains are organized in primary, secondary and tertiary structures, with a final shape of fibrils [53], as shown in Figure 1. 

The different types of collagen are classified depending on domain structure and their suprastructural organization. Each collagen type has a specific alpha chain with its own domain structure which contributes to the classification by collagen type. 

Collagens can be fibril-forming (i.e., collagen types I, II, III, V, XI, XXIV, XXVII), fibril-associated with interrupted triplex helix (i.e., collagen types IX, XII, XIV, XX), fibril-associated with interrupted triplex helix-like (i.e., XVI, XIX, XXI, XXII), and network-forming (i.e., the basement membrane collagen IV, beaded filament-forming collagen VI, anchoring fibril-collagen VII, hexagonal network-collagens VIII and X, transmembrane collagens XIII, XVII, XXIII and XXV, multiplexin collagens XV and XVIII, and other molecules with collagenous domains named collagens XXVI, XXVIII). Collagens in each class have their own specialized function and contribute to higher order tissue structures.

Collagen is the main component of membranes in most living organisms and has a structural function, as in bones and cartilages [54]. Slight differences in the composition of the triplet units determine differences in collagen types. According to the structure and supramolecular organization of the 28 known collagen types to date [53], they also have different allocation in mammalian tissues shown in Table 2; type I collagen accounts for 80%–85% of the collagen in the body.

Partial hydrolysis of collagen followed by thermal treatment, produces gelatin with molecular weights from 3 to 200 kDa as shown in Figure 1, depending on the raw material used and handling conditions [55]. In particular, depending on the process used, two types of gelatin, namely type A (acid hydrolysis) and type B (alkaline hydrolysis) are generally obtained [56]. During hydrolysis, the natural molecular bonds between individual collagen strands are broken down into a form that rearranges more easily. Therefore, gelatin is a mixture of single or multistranded polypeptides, each with extended left-handed helix conformations and containing 50–1000 amino acids; its chemical composition is closely related to that of collagen. Gelatin contains many glycine residues (almost one in three residues, arranged every third residue), proline and 4-hydroxyproline residues. A typical structure is -Ala-Gly-Pro-Arg-Gly-Glu-4Hyp-Gly-Pro-. The approximate amino acid composition of gelatin is: glycine 21%, proline 12%, hydroxyproline 12%, glutamic acid 10%, alanine 9%, arginine 8%, aspartic acid 6%, lysine 4%, serine 4%, leucine 3%, valine 2%, phenylalanine 2%, threonine 2%, isoleucine 1%, hydroxylysine 1%, methionine and histidine < 1% and tyrosine < 0.5%. These values vary, especially the minor constituents, depending on the source of the raw material and processing technique [57].

Collagen represents an excellent source of peptides with biological activities [58], obtained by several processes including chemical hydrolysis, enzymatic treatment, and fermentation with proteolytic bacteria. Enzymatic hydrolysis with appropriate proteolytic enzymes seems to be the most effective approach for the bioactive peptides generation [59]. It can be achieved under controlled conditions by specific proteases to obtain reproducible collagen hydrolysates. Moreover, the use of the mixture of several proteases and sequential enzymatic hydrolysis by enzymes with different specificities is also recommended to enhance collagen hydrolysis. Peptides in collagen hydrolysates (300–8000 Da) are subsequently fractionated by ultrafiltration and several chromatographic techniques [59]. Incubation time and the concentration of enzymes greatly influence the average molecular weight of obtained peptides which subsequently impacts their activities; a high degree of enzymatic hydrolysis with the release of small peptides produces collagen hydrolysate with interesting bioactivities [58].

## 4. Extraction of Marine Collagen

Depending on the marine sources, different techniques have been proposed to obtain collagen. Nevertheless, it is possible to establish a general methodology to isolate collagen from fish by-products and other marine sources, based on three steps: preparation, extraction and recovery.

The preparation involves cleaning, the separation of animal parts, and size reduction by cutting or mincing the samples. Generally, fish are split up in skins, scales, fins and bones, because their collagen composition is different (e.g., mineralization in fish bones and scales). In the case of jellyfish, it is common to separate the oral arms from the umbrella and to then divide the umbrella into the mesoglea, exumbrella and subumbrella [28].

Size reduction of these samples is essential to facilitate subsequent chemical (pre)treatment actions, used to remove noncollagenous proteins, pigments or fats. The common method provides the use of a basic pretreatment with sodium hydroxide (NaOH), which does not cause structural modification to collagen chains, alcohols (namely butyl-alcohol or ethanol), and oxygen peroxide in the removal process of noncollagenous proteins, fats and pigments, respectively [16,18,19,60,61,62]. Furthermore, to remove noncollagenous proteins from codfish skin, the use of sodium chloride (NaCl) as an alternative to NaOH was also proposed [63].

Moreover, to improve the collagen extraction from bone, cartilage and scales, ethylenediaminetetraacetic acid (EDTA) is recommended for demineralization purposes [15,23,64]. Alternatively, HCl can also be used [65].

For the extraction phase, it is well-known that the solubility of collagen in cold water is poor because of the presence of strong cross-links in its triple helix structure. There are two different conventional methods largely used: extraction of acid-solubilized collagen, and extraction of pepsin-solubilized collagen. Using these two methods, yield, chemical composition, and characteristics of the extracted collagen differ from one another. The whole extraction phase is performed at 4 °C.

When collagen extraction is performed using only acid, the product is referred to as acid-soluble collagen (ASC). For collagen extraction from marine animal tissues, acetic acid is the most used dilute acid (generally, at the final concentration 0.5 M), but also citric acid and lactic acid are utilized. The extraction protocols are generally adapted from methods reported by [23,30].

About 95% of marine invertebrates such as jellyfish consists of water, which affects collagen solubility in acetic acid. Therefore, homogenizing or freeze-drying jellyfish is necessary to improve the collagen solubility in diluted acids and, accordingly, increase the extraction yield.

Recently, Yusoff et al. proposed a new method to extract collagen from aquatic animals, in which the acidic treatment is combined with a sequence of physical and mechanical treatments, including pH adjustments, homogenization, mixing, as well as sonication [66]. By increasing physical intervention in jellyfish, the extraction yield increased significantly compared to the conventional extraction processes [49].

When the enzyme pepsin is added in the extraction process, the extracted collagen is referred to as pepsin-soluble collagen (PSC). This treatment is very useful, since proteases cleave telopeptide cross-linked regions without breaking the integrity of the triple helix, and thus hydrolyze some noncollagenous proteins and increase the purity of collagen [67]. Therefore, in most cases, enzymes are used to obtain specific protein products, high yield and reduced wastes, as well as a decrease in the antigenicity caused by telopeptides [68,69,70]. However, when a high amount of pepsin is used for long time, PSC yield may be lower because the collagen is likely cleaved, impairing the triple helix’s integrity [62].

During the recovery step, collagen is precipitated, generally by adding NaCl to a final concentration 2.3–2.6 M. The resultant precipitate is collected by centrifugation, dissolved in 0.5 M acetic acid, dialyzed and freeze dried [71].

From jellyfish, collagen is generally extracted by a methodology based on solubilization in a 0.5 M acetic acid solution (typically for three days), followed by salting-out by dialysis against a Na_2_HPO_4_ solution. The precipitated collagen is separated by centrifugation, solubilized in acetic acid, and purified by reprecipitation by adding solid NaCl at a concentration of 0.9 M. ASC can also be digested with pepsin to obtain atelo-collagen [27,72].

The intact collagen fibrils of sea urchins’ peristomial membranes are different from other collagens and cannot be extracted by traditional methods of acid solubilization, as this method generally produces it in a hydrolyzed jelly form [73]. Therefore, the minced native tissue is sequentially treated with a hypotonic solution and an SDS-based decellularizing solution to remove both cell debris, skeletal parts and pigments [73,74]. After 3–4 days in the disaggregating β-mercapto-ethanol solution, the collagen fibers obtained are then removed by a filtration step and dialyzed against a 0.5 M EDTA-Na solution. The same protocol is used to extract collagen fibers from starfish aboral arm walls, but the samples are processed with an additional step in 1 mM citric acid between decellularizing and disaggregating solutions to remove the calcium carbonate ossicles present in the fresh tissue [75].

## 5. Marine Collagen Proceeding

Marine collagen is used in its native fibrillar form as well as after denaturation. During biosynthesis, collagen acquires a number of post-translation modifications that are critical to structure and biological functions of this protein [76]. The extent of these modifications influences not only the collagen extraction, but also the denaturation and consequently, the processing of the protein [12]. In particular, the denaturation temperature increases as a result of an increase in the hydroxyl groups of hydroxyproline and hydroxylysine obtained by these modifications. The content of hydroxyproline and hydroxylysine depends on different marine species and on the water temperature of their native habitat, and the content is lower than vertebrate collagens [77]. Denaturation offers the possibility to fabricate several collagen forms, including sheets, tablets, pellets, and sponges. When the triple helix structure of collagen is broken into single-strand molecules by acid, alkaline or enzymatic hydrolysis, a water-soluble gelatin is obtained. Gelatin can be easily processed using thermoplasticization techniques by applying heat and mechanical stresses in extrusion-based technologies.

Gelatin may be chemically treated to bring significant changes in its physical and chemical properties. Typical reactions include acylation, esterification, deamination, cross-linking and polymerization, as well as simple reactions with acids and bases [78,79].

The formation of thermo-reversible gels is obtained when the aqueous solution of gelatin with a concentration greater than 0.5% is cooled to approximately 35 °C–40 °C. The rigidity or strength of the gel depends upon gelatin concentration, structure and molecular mass, pH, temperature, and presence of any additives [80,81].

Two common technologies are employed to process collagen, gelatin and other general proteins: wet (or solvent) and dry processes. The wet process is based on the dispersion or solubilization of collagen proteins in a solvent medium followed by the solvent removal, which can occur by drying or through a solvent–nonsolvent exchange mechanism. Most of the literature works on the use of collagen and gelatin to prepare films, fibers or sponges are based on wet processes [82]. In the dry process, mainly employed for other kind of proteins, thermal and mechanical energies can be used to disrupt intra and intermolecular interaction of biopolymers by extrusion or mechanical mixing, common plastic processing techniques. A large variety of extrusion-based processes can therefore be employed, including film casting, film blowing, compression molding, extrusion foaming and fiber spinning.

In almost all cases, the manufacturing of protein-based products in both wet and dry processes requires the use of plasticizers that improve both the processing and properties of the materials. Plasticization, often obtained with mixtures of plasticizers, leads to materials with lower glass transition temperatures, lower elastic modulus and higher deformability. Depending on the concentration of plasticizers, it is also possible to control the viscoelastic properties of a melt, which is necessary to optimize the shear or the elongational rheology during dry processes.

### 5.1. Dry Process

Due to the strong intermolecular and intramolecular hydrogen bonds, native proteins cannot be processed like common thermoplastic polymers. However, in presence of suitable plasticizers, they can melt and flow if treated at higher temperatures (usually 60 °C–160 °C) and under shear. In this condition, proteins are denaturated and the melt is processed in conventional manufacturing technologies employed for thermoplastics, like extrusion, injection, and compression molding, blowing and foaming.

Plasticizers have different roles: they (i) contribute to breaking hydrogen bonds replacing inter-and intra-molecular interactions with protein and plasticizers interactions, (ii) lower the melt temperature below the decomposition temperature of the protein, (iii) reduce the viscosity of the melt. The process of increasing the macromolecular mobility (which leads to materials with lower melt temperature and higher flexibility by using plasticizers) is common in conventional polymers and is known as “plasticization”.

In general, plasticizers are low molecular weight molecules compatible with protein, and are therefore usually polar and hydrophilic. In particular, the ability of the plasticizer to interact with protein is fundamental and its efficiency depends on several factors, including its size (molecular weight), shape and functionality (mostly, number of oxygen atoms) [83].

The most common plasticizers for proteins are water, monosaccharides, oligosaccharides, polyols, lipids and derivatives [84]. Glycerol is the most used plasticizer in thermoplastic processing of proteins, e.g., corn gluten meal, wheat gluten, soy protein, zein, and kafirin [85,86,87].

### 5.2. Extrusion-Based Processes

Extrusion is a highly efficient and continuous process usually employed for large-scale manufacturing and used for thermoplastic polymers. The materials are continuously introduced into a hopper, conveyed by a screw, and pushed through a die of desired shape. During the conveyance, several operations can be performed: heating, cooling, feeding of solids and liquids, conveying, compressing, shearing, reacting, mixing, melting, homogenizing, cooking, and shaping. 

**Figure 2:**
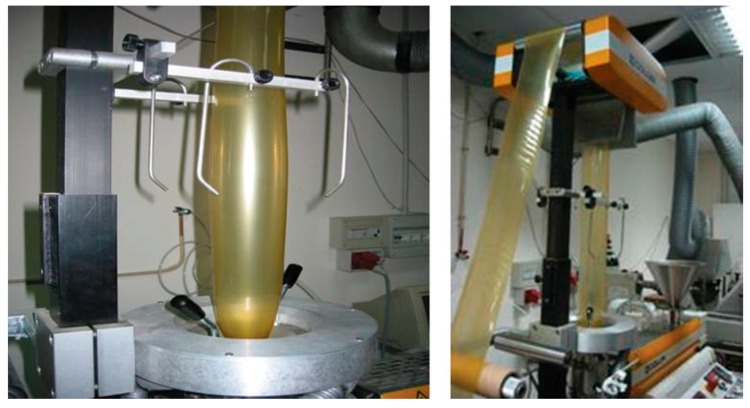
Lab-scale film blowing of thermoplastic zein. Reproduced with permission from [91]. Copyright publisher, 2020. In the figure, the film bubble during the film-blowing process of thermoplastic zein is shown. The zein powder was first plasticized with poly (ethylene glycol) 400 directly in the extruder, without the use of a solvent and of a premixing phase.

So far, there is no literature available regarding the extrusion process of thermoplastic gelatin, but there are some reports available for plant protein-based films, such as zein and gluten [88,89] that can be used as a point of reference for the development of novel products and processes for marine collagen. Zein sheets plasticized with fatty acids were produced by extruding a moldable, dough-like resin prepared by precipitating zein and oleic acid from aqueous-alcohol solutions [90]. Oliviero et al. showed that it is possible to prepare thermoplastic films from commercial zein/PEG400 mixtures by film blowing technology, as shown in Figure 2 [91]. This gives films a low-enough thicknesses and suitable mechanical properties for packaging applications, making them comparable to those from common synthetic polymers. 

Thermoplastic processing of gelatin was first reported in 2007 [92]. Thermoplastic formulations containing glycerol (20, 25 wt%) and lactic acid (20 wt%) were prepared using a twin counter rotating internal mixer at 60 °C, for 6 min at a speed of rotation of 60 min^–1^. Films with different mechanical properties were then obtained by compression molding. There are not works available in the scientific literature regarding the use of other manufacturing technologies for film production, such as film casting or film blowing.

**Figure 3:**
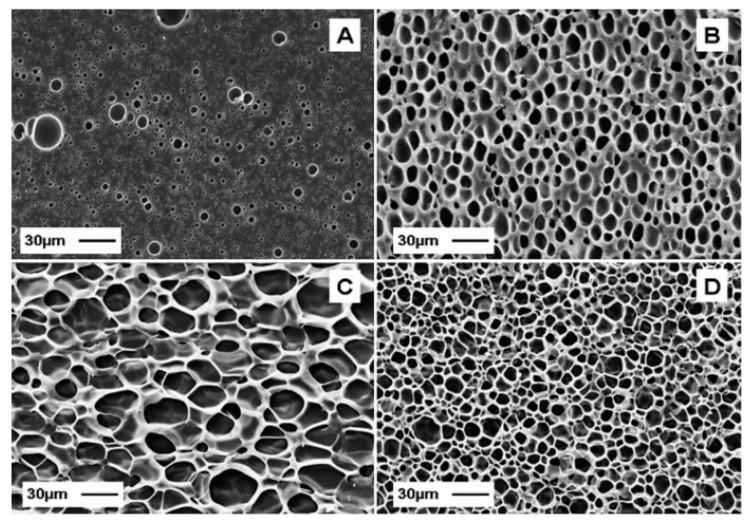
Micrographs of thermoplastic gelatin foams. Scanning electron microscopy (SEM) micrographs of thermoplastic gelatin foamed with N2-CO2 80-20 vol.% at Psat = 180 bar, PDR = 700 bar/s and TF = 44 °C (**A**), 80 °C (**B**), 120 °C (**C**) and 140 °C (**D**). Reproduced with permission from [92]. Copyright publisher, 2020. The gelatin powder was first plasticized with glycerol directly in the mixer.

Thermoplastic gelatin can be successfully foamed by employing a mixture 80/20 of N_2_/CO_2_ at temperature above its glass transition temperature of 50 °C [92]. As shown in Figure 3, good foams with different cellular morphologies can be obtained in the temperature range of 80 °C–140 °C. 

As a major drawback, neat thermoplastic gelatin has a strong sensitivity to moisture, which can heavily decrease its barrier as well as its thermomechanical properties. Blending with hydrophobic biodegradable polymers has been a typical approach already employed for other natural occurring polymers such as in the case of thermoplastic starch, or other thermoplastic proteins [93,94]. Blends of thermoplastic gelatin with poly(butylene succinate), a polyester deriving from butanediol and succinic acid have been investigated with the aim to improve not only the resistance to moisture, but also the rheological properties, as well as the diffusivity and solubility of the blowing agent for the foaming technology [95]. Also, in blending with PCL, it is possible to foam the thermoplastic gelatin. In that case, a selective extraction of the water-soluble gelatin phase allowed for the development of porous network pathways characterized by multimodal porosities for biomedical applications [96,97,98].

## 6. Biomedical and Pharmaceutical Applications of Collagen

Numerous attempts have recently been made to use marine collagen as a biomaterial. Collagen is the most promising natural biomaterial as scaffold in tissue engineering, as it is abundant, biocompatible, biodegradable, resembles the components present in the extracellular matrix and supports the connective tissue including skin, tendon, bone, cartilage, blood vessel, and ligaments [37]. However, collagen from fish is less crosslinked and its mechanical strength is poorer than collagen extracted from bovine. Therefore, the use of marine collagen as biomaterial in tissue engineering is possible after crosslinking treatment [72,99]. In order to improve the mechanical strength of marine collagen, some kinds of bioceramics with the similar constituent to the intrinsic inorganic components of nature bone are also widely used in collagen scaffolds for bone regeneration. Apart from the enhancement of mechanical properties, they can also improve osteoconductive ability, dimensional stability and increase the surface area for cell attachment on the composite scaffolds [100].

Marine collagen is also widely used in dentistry [12,101], generally as membrane, bone graft materials, an agent for local delivery and a hemostatic agent. In particular, the membranes are used in periodontal and implant therapy to guide soft tissue regeneration and inhibit the rapid regrowth of skin when implanting bone which takes longer to generate. Most collagen membranes on the market are resorbable in a few days.

The collagen used for local drug delivery is generally in the form of membranes. The membrane most used is constituted by two components: chlorhexidine and collagen [102]. Another product used for local drug delivery comprises tetracycline fibers impregnated in collagen fibers. Both systems allow diffusion of the drug as collagen undergoes resorption, releasing the drug from the matrix in a controlled manner [103].

Marine collagen is also used to control bleeding. Different products are commercially available; they have sponge-like structures and are highly absorbent and able to hold many times their own weight of fluid. They may be cut to desired shape and applied to a bleeding surface. The placed product rapidly absorbs the blood, creating an artificial clot-like structure that stop the bleeding at the site. These products resorb completely within 14 to 56 days [104].

The hydrophilic nature and the molecular structure of collagen, characterized by a high content of diaminodicaroxylic amino acids and carbohydrate, provide a surface geometry very suitable for cell adhesion and wound repair in dentistry and surgery. Another factor, which promotes the adhesion of fibrogenic cells on collagen implants, is the presence of a glucoprotein-like fibronectin on cells surface that have a high affinity with particular regions of collagen surface [104]. There are two primary forms of collagen wound dressing: sponges and films. Sponges are used as both temporary and permanent coverings, and the cellular growth depends on the sponge porosity and the presence of fibrous structure. The films are instead produced by casting on methacrylate surfaces, and are crosslinked by using UV radiations in order to improve their handling properties [104].

The use of collagen-based materials in drug delivery involves the study of different aspects, such as in vivo instability, bioavailability, solubility and body tissue absorption with target-specific delivery and tonic effectiveness. To this purpose, nanotechnologies play an important role in order to develop new drug delivery systems for targeting drugs to specific body parts [105].

A collagen skeleton of marine sponges was used to develop new bio-based topical formulations, where the marine collagen acts as bioactive, biomimetic carrier for the loading of L-cysteine hydrochloride, facilitating the wound healing processes due to its glycosaminoglycans [11].

In addition, due to its abilities in skin repair as shown when tested on female volunteers [106], marine collagen has been increasingly utilized for the development of cosmeceutical products containing bioactive ingredients with pharmaceutical benefits. Cosmetic products, based on marine extract collagen, have shown a comparable effect on the skin with that of animal collagen in terms of pH, moisture and sebum. Moreover, marine collagen hydrolysate has shown an effect on the inhibition of photo-aging [107]. The hydrolysis of polypeptides with a small molecular weight, capable of penetrating the skin, could also overcome a major issue related to fish collagen usage, i.e., the low denaturation temperature that is much lower than the temperature of the human body. In particular, we focus our attention on the applications of collagen extracted from fish waste, echinoderms, and jellyfish.

### 6.1. Collagen and other Collagen-Derived Peptide Bioactivities from Sustainable Marine Sources

Several studies have been performed to define potential biomedical and pharmaceutical applications of marine collagen and collagen-derived peptides as well as gelatin from sustainable sources. Some possible uses are reported below, and the applications are schematized in Figure 1.

#### 6.1.1. Fish Waste

Collagen derived from fish waste and bioactive peptides derived from collagenous sources have been shown to have several bioactivities with possible cosmeceutical and pharmaceutical applications, as well as biomaterial for tissue engineering [12,58]. Collagen extracted from codfish and salmon skins was characterized to evaluate its inclusion as a component in cosmetic formulations [108]. It demonstrated a good capacity to retain water, which shows promise as a suitable candidate for dermal moisturizing applications. In addition, possible skin irritation and inflammation potentially due to the extracted collagen was evaluated by using a human 3D reconstructed epidermal model (In Vitro Epiderm™ Assay Kit, EPI-200, MatTek Corporation) and by measuring inflammatory mediators (i.e., IL-6 and IL-18) by commercially available ELISA kits. Results showed that fish collagen did not have any irritation or inflammatory effects.

A pilot study obtained collagen oligopeptide-rich hydrolysate from codfish skin by using a collagenolytic protease from the deep-sea bacterium *Pseudoalteromonas* sp. SM9913 [109], and bioactivity testing showed that obtained collagen peptides had antioxidant activity, reducing free radicals at 10 mg/mL, higher than those of hyaluronic acid, an ideal material in cosmetics. The obtained hydrolysates (in which collagen peptides accounted for approximately 95%) also had promoting cell-proliferation effects on human dermal fibroblasts and showed no toxicity. Acute toxicity was tested on Kunming mice, while skin irritation was tested on rabbits [109]. The in vivo experiments showed that there was no depression, hair loss, dyspnea, wound formation, significant differences in body weight or pathological abnormalities in various body parts of the mice, and edema, erythema, and rough or thinning skin were not observed in the rabbits.

In vivo studies in the mice showed that salmon skin collagen peptides reduced oxidative damage [110] and acetylcholinesterase (AChE) and increased phosphorylated cAMP-response element binding protein (p-CREB) and brain-derived neurotrophic factor (BDNF) expression [111]. Moreover, collagen from salmon skin was used with hydroxyapatite to produce scaffolds for bone regeneration, based on the biomimetic mineralization principle [112]. The scaffold with interconnected pores allowed human mesenchymal stem cells to adhere and proliferate, providing a good support for osteogenic differentiation.

Subcritical water-hydrolysed fish collagen peptide (SWFCP) from tuna skin shown adipogenic regulatory activity [110,113]. In particular, SWFCP downregulated the expression of key adipogenic target genes (C/EBP and PPAR protein) and transcription factors in 3T3-L1 preadipocytes exposed to dexamethasone and insulin. SWFCP also downregulated the expression of aP2, an adipogenic target gene, hence inhibiting adipogenic differentiation. Furthermore, SWFCP also reduced lipogenesis in hepatocytes and significantly affected other obesity-related factors, e.g., low serum cholesterol, low serum triglyceride, and low-density lipoprotein, and reduced the size of epididymal adipocytes. 

Collagen hydrolysates were also obtained from unicorn leatherjacket skin (*Aluterus monoceros*) by using collagenase at three different temperatures 5 °C, 25 °C and 50 °C (collagen peptides named CP-5, CP-25 and CP-50, respectively) [114]. Results showed that CP-5 was more active than the others for anticancer, antidiabetic and wound healing activities, suggesting that collagen extraction and hydrolysis parameters can influence the bioactivity. 

Finally, several sharks are landed as secondary catches from the fishing of other species. Various parts of the body have found a plethora of applications on the market. Examples are the meats as food, the cartilage to produce chondroitin sulphate (food supplements for treating osteoporosis and cancer), squalene for skin care, and skin for shoes and handbags. In addition, shark skin is very rich in collagen type I, while shark cartilage is rich in collagen type II. Collagen from shark tissues have been used to prepare a gel matrix for in vivo culturing of fibroblasts as a food supplement to create functional foods, and to also increase the cryoprotection of foods [115]. Moreover, the antioxidant capacity of type II collagens (i.e., type II ASC, CIIA and PSC, CIIP) was also assessed by 1,1-diphenyl-2-picrylhydrazyl (DPPH) radical scavenging capacity, showing that the reducing power of CIIP was greater than that of CIIA [116].

Collagen type I was extracted from the skin of the common smooth-hound, *Mustelus mustelus* (Linnaeus, 1758) and combined with chitosan to produce a composite film in order to test it as green bioactive film to preserve nutraceutical products [117]. It was found that this combined film had lower tensile strength and higher elongation at break when compared to chitosan film, and lower water solubility and lightness when compared to collagen film. In addition, this collagen-chitosan-based biofilm showed antioxidant activities evaluated by using the DPPH assay, as well as potential UV barrier properties.

Marine collagen from fish and especially marine collagen-based scaffolds have proved to be useful alternatives for several biomedical applications [12]. For example, collagen from chum salmon, *Oncorhynchus keta* (Walbaum, 1792) skin increased serum osteocalcin, size, mineral density, and dry weight of femurs in growing male rats [118]. Oral administration of collagen from skins of Gadiformes fish species controlled cartilage degradation in osteoarthritis-induced rabbit models [119].

Tilapia collagen/bioactive glass (Col/BG) nanofibers, fabricated via electrospinning, were used as wound dressing to protect against infection and to promote wound healing and skin regrowth in both *in vivo and in vitro studies* [120].

Type I collagen from the Nile tilapia, *Oreochromis niloticus* (Linnaeus, 1758) scales promoted rat odontoblast-like cells and accelerated matrix mineralization [121], which indicated that it could be an alternative to type I collagen from mammals in the application for tissue regeneration in oral-maxillofacial area.

Marine collagen was recently studied as a promising biomaterial with great potential in drug delivery applications due to its unique properties. A scaffold-controlled release system for skin tissue engineering, based on poly (D,L-lactide-co-glycolide acid) (PLGA) microspheres and fish collagen, chitosan and chondroitin sulfate scaffolds were obtained by freeze-drying. The developed marine collagen drug delivery system exhibited a tunable protein release rate, depending on the ratio of fish collagen, and it showed good biocompatibility and ability to promote fibroblast cell proliferation and skin tissue regeneration [122].

#### 6.1.2. Echinoderms

There are particular collagens from echinoderms that have been described, such as those from sea urchin and starfish by-catches. They are well-known for their unique connective tissues, named mutable collagenous tissues (MCT), recently proposed as possible inspiration for “smart dynamic biomaterials” for tissue engineering and regenerative medicine applications [74,123]. It can be used to develop collagen barrier-membranes for guided tissue regeneration (GTR) [75].

Echinoderm-derived collagen membranes (EDCMs) are similar to each other in terms of structure and mechanical performances, and are much thinner and mechanically more resistant than the commercial membranes, suggesting that they could be alternative collagen sources for the production of efficient GTR membranes.

Particularly, the sea urchin peristomial membrane, a well-known MCT, is a food industry waste that can be transformed into a highly valuable by-product. It has been suggested as sustainable and eco-friendly source of fibrillar collagen to produce membranes for regenerative medicine applications [24].

#### 6.1.3. Jellyfish

Recently, the jellyfish *R. esculentum* has received some attention as the type I collagen extracted from this species is quite similar to the human type, making it suitable for different applications in the biomedical field [10]. It was cross-linked with 1-Ethyl 3-(3-Dimethylaminoprophyl)-Carbodiimide (EDC) to form collagen-based sponges which showed hemostatic properties as blood clotting after tail amputation in rats, suggesting that it may be a suitable candidate for hemostatic material and wound-dressing applications [31]. Moreover, peptides derived from *R. esculentum* collagen were involved in wound-healing processes in vivo, by increasing the production of chemotactic factors TGF-β1 and β-FGF [124].

The capacity of the collagen to promote cell migration in wounded tissue suggests its possible use in synthetic matrix production for cartilage tissue engineering, mainly fibrous, hydrogel or hybrid materials [125,126]. Several efforts have been focused on collagen-based material colonization with cells of connective tissue such as fibroblasts or endothelial cells and other growth supports, in an attempt to produce a similar biocompatibility and immune response with collagen-based material commercially available. Recently, the type I collagen extracted from the blubber jellyfish, *Catastylus mosaicus* (Quoy and Gaimard, 1824) effectively supported preosteoblast growth [127].

Collagen from the giant Nomura’s jellyfish, *Nemopilema nomurai* mesoglea was used to prepare porous collagen scaffolds. This collagen did not show any cytotoxicity and therefore resulted biocompatible with primary human fibroblasts (HFs) and endothelial cells. It showed a better cell viability compared with bovine collagen, glucan, gelatin and hyaluronic acid, while the in vivo implantation produced a similar immune response to commercial bovine-derived collagen [72]. Moreover, *N. nomurai* collagen was used to produce a hybrid collagen/hyaluronic acid 3D highly porous scaffold, which allowed fibroblast proliferation on its wide surface without interfering with cell viability [99].

An in vivo comparative study of jellyfish and bovine sponges as prototype medical devices reported that jellyfish collagen is able to stimulate both transcription and translation, thus enhancing immunoglobulin and cytokine production [50]. Results confirmed an immunological response of jellyfish collagen sponges, comparable to that stimulated by bovine collagen and gelatin [50].

Furthermore, *R. esculentum* collagen was used for growing human and rat nasal septal chondrocytes, with no cytotoxic effects and good biocompatibility, confirming the marine collagen as a suitable candidate for cartilage bioengineering [128]. Recently, *R. pulmo* type II collagen was used to develop a collagen-based biomaterial. The scaffold was implemented with nanoreservoirs of the growth factor TGF-β3 and human stem cells, building up a new adaptable device for articular cartilage repair [129].

The immunostimulatory effect of *N. nomurai* collagen stimulated the production of immunoglobulin and cytokine, not only in the specific human hybridoma cell line HB4C5, but also in the peripheral blood lymphocytes (PBL). In addition to these effects, the tumor necrosis factor (TNF)-α and the interferon (IFN)-γ levels were increased in PBL [130].

Thrombosis and hypertension are among the main causes of cardiovascular-associated death [131,132], thus the research for new treatments still remains an active field. In this regard, *R. pulmo* collagen was used to fabricate an apta-sensor for the clinical detection of thrombin in the blood. The collagen was cross-linked to the designed amine thrombin aptamer using glutaraldehyde. This hybrid sensor displayed a detection limit of 6.25 nM, largely below the imposed clinical limits, suggesting interesting future implication of collagen as a promising candidate for clinical analysis of thrombin [133].

Recently, Liu et al. described four novel angiotensin-converting enzyme (ACE) inhibitory peptides purified from *R. esculentum* collagen hydrolysate [134]. After the jellyfish collagen peptides (JCP) oral administration, the angiotensin II concentrations in the kidney decreased, leading to a significant decrease in both systolic and diastolic blood pressure [135].

Jellyfish collagen is a source of a great number of antioxidants. Recently, it was demonstrated that peptide fractions from *R. pulmo* collagen were able to prevent oxidative stress in HEKa cells treated with H_2_O_2_ [136]. Moreover, collagen peptides exhibiting scavenging and antifatigue activities were identified in *R. esculentum* [137,138], as well as collagen hydrolysate with several activities (including superoxide anion-scavenging and melanogenesis-inhibitory activities) based on the capacity of the hydrolysate to chelate copper inhibiting the intracellular tyrosinase activity [139]. Both jellyfish collagen and its hydrolysate were found to operate as UV radiation protectors, proposing their possible utilization in skin care industries [107]. Similarly, it was demonstrated that collagen peptides from *S. meleagris* are an effective tyrosinase inhibitor, acting on glutathione (GSH) levels [140].

## 7. Other Potential Applications of Marine Collagen: Food Additives and Packaging

Other than in the biomedical and pharmaceutical fields, collagen can find wide application in the food industry, as a food additive or packaging. However, so far, there is no literature available regarding the use of marine collagen in these applications, but there are many reports available for bovine, porcine, ovine and duck feet collagen. In this section, some of these examples are reported as references for the development of novel products and applications of marine collagen.

Currently, collagen has become a necessary ingredient toward the healthy food development. The production of collagen in the body decreases with age and with an unhealthy diet. Therefore, collagen has been added to a variety of foods [2]. Collagens are usually used as food additives to improve the rheological properties and reduce the fat consumption of sausages and frankfurters. Collagens are used also to ensure the presence of adequate amount of animal nutritive fibers [141].

Collagen-based edible films and coatings have already been proposed to protect, maintain and extend the shelf life of different food products. The film or coating acts, in this case, as a barrier layer against the migration of oxygen, moisture and solutes, providing structural integrity and vapor permeability to the food product [142]. Moreover, it prevents fat oxidation, discoloration, microbial growth and preserves the sensory qualities. 

If properly treated with solvents, the collagen from the corium layer of food grade beef hides can be used to produce sausage casings (preformed casings) wherein subsequently the meat batter is stuffed [143]. In addition, collagen casings can be also coextruded around sausage meat batter, obtaining a process that is continuous and well-controlled [143]. However, in this coextrusion process, collagen for its complex structure must be first treated in order to obtain a dough or a suspension to be fed to the extruder. Conversely, gelatin, the denatured form of collagen, is easily processed by using thermoplasticization techniques by applying heat and mechanical stresses in extrusion-based technologies. A plasticizer is needed as it acts as an internal lubricant, leading to an increase of molecular mobility, necessary to promote the melt flow. The gelatin-based films obtained in this way are transparent and have excellent barrier properties against oxygen. 

However, as a major limitation to widespread use, they have a strong sensitivity to moisture, which is responsible for a drastic reduction in barrier and as thermomechanical properties. One strategy to overcome this weakness is to associate the gelatin based film with a moisture resistant biodegradable polymer through laminating (coextrusion) as is typically done in film packaging, where multilayered structures that consist of distinct layers of moisture and oxygen barriers have been optimized for the specific different package and conditions [144]. 

A biodegradable three-layer gelatin film was obtained by hot compression of sodium montmorillonite-plasticized gelatin as the inner layer and cross-linked dialdehyde starch and plasticized gelatin films as the outer layers [145]. The multilayer film displayed a compact and uniform microstructure due to the highly compatible individual layers which could interact by strong hydrogen bonding. Lamination reduced moisture absorption compared to the single layers, while maintaining transparency and biodegradability [145]. Films of plasticized gelatin with 30% (*w/w*) glycerol can be also combined with poly (lactic acid) films as outer layers. The obtained multilayer film shows water vapor permeability values of around 1.2 × 10^−14^ kg m s^−1^ Pa^−1^ m^−2^ [146], which is higher than that obtained from other commercial polymers such as high density polyethylene or poly (vinyl chloride) (2.4 × 10^–16^ and 0.7–2.4 × 10^–16^ kg m s^−1^ Pa^−1^ m^−2^, respectively) [147]. Poly (lactic acid) has been also used for gelatin-based three-layer film manufacturing with the dip-coating technique [148]. 

Further factors that should be considered when designing a food packaging are chemical nature of food, controlled release mechanisms, food organoleptic characteristics, additive toxicity and storage [149]. Consequently, different types of additives must be used to achieve suitable gelatin-based films or coatings for food packaging. Much research has been conducted to develop active packaging films and coatings, including antimicrobial, antioxidant and other agents which can enhance the biological features of foods. Recently, natural additives without negative effects on human health have been studied in order to reduce the use of synthetic chemical additives in the food industry. These additives can be obtained from different sources, including plants, animals, bacteria, algae, fungi and by-products generated during fruit and vegetable processing. Bioactive peptides, such as lysozyme, can be incorporated into gelatin films for food preservation. In particular, lysozyme incorporated into fish gelatin films did not inhibit the growth of *Escherichia coli*, but is effective against Gram-positive bacteria at very low concentrations [150]. The antibacterial activity of fish skin gelatin films incorporated with various concentrations (10, 20 and 30 wt%) of peppermint and citronella oils were also studied [151]. A growth inhibition of *E. coli* and *Staphylococcus aureus* was achieved with a success of higher than 80% with each oil loaded at 10 wt% [151]. The incorporation of chitosan into gelatin film forming solutions also resulted in active films against relevant food poisoning micro-organisms. These authors observed that mixing gelatin with chitosan was a means to improve water and mechanical resistance of gelatin films, but also to provide gelatin films with antimicrobial activity [152]. Wu et al. developed fish gelatin films incorporated with nanocapsules containing cinnamon essential oil with the purpose of improving and controlling their release rate [153]. The results of antimicrobial test showed a higher inhibition zone for the obtained film with cinnamon essential oil nanoliposomes compared to that of gelatin with cinnamon essential oil, demonstrating an improvement in antimicrobial stability along with a decrease in release rate after storage for one month. Liu et al. investigated the applicability of gelatin-based films packaged with sunflower oil [153]. An improvement in antioxidant activity was demonstrated over a long period of storage (six weeks), as well as the preservation of the functional properties of the new films. Food products such as fish, meat, fruits and vegetables can be coated with gelatin-based films in order to retard degradation processes due to the transport of gases (O_2_ and CO_2_) and water vapor. However, new methods and formulations for the production of marine gelatin-based films with improved final properties and potential applications require further exploration.

## 8. Conclusions

Contemporary societies across the world are facing the urgent need to find alternative, sustainable and eco-friendly resources due to the overexploitation of terrestrial resources and the problem of waste disposal. At the present time, humans live longer than their ancestors, which means that they need more support from the pharmaceutical, nutraceutical and biomedical fields to age better. Hence, research has been focusing on marine organisms to find new and alternative sources of natural compounds [154]. In the latest 20 years, more than 28 marine natural products and 175 chemical entities were found, and hundreds of new compounds are still being discovered every year, likely due to the advances in collection and molecular biology techniques [155,156]. To date, there are seven approved marine-derived drugs in clinical use, and about 26 natural products in phase I to phase III clinical trials [154].

Collagen has several applications in different fields, including nutraceuticals, cosmeceuticals, biomedicals, biomaterials and the food industry. Such a large variety of applications means that collagen can be key for the health and well-being of humans. To date, the sources of collagen mainly relied on terrestrial organisms, but they are becoming limited due to the spread of diseases and increasing alternative dietary choices of humans. This review highlights how marine organisms and their wastes can be a sustainable, eco-friendly source of collagen for the applications aforementioned. 

## Figures and Tables

**Table 1:** Collagen content in fish, sharks and scyphomedusae. The collagen content is expressed as percentage of wet or dry (*) mass.

Species	Tissue	Collagen Content	Reference
Pisces			
*Priacanthus tayenus* (Richardson, 1846)	bone	1.6	[15]
	skin	10.9*	[15]
*Hemibragus macropterus* (Bleeker, 1870)	skin	28.0*	[16]
*Syngnathus schlegeli* (Kaup, 1856)	skin	33.2*	[17]
*Lagocephalus gloveri* (Abe and Tabeta, 1983)	skin	54.3*	[18]
*Takifugu rubripes* (Temminck and Schlegel, 1850)	skin	44.7*	[19]
*Saurida* spp.	scales	0.79	[20]
*Trachurus japonicas* (Temminck and Schlegel, 1844)	scales	1.5	[20]
*Mugil cephalis* (Linnaeus, 1758)	scales	0.4	[20]
*Cheilopogon melanurus* (Valenciennes, 1847)	scales	0.7	[20]
*Dentex tumifrons* (Temminck and Schlegel, 1843)	scales	0.9	[20]
Mollusca			
*Illex argentinus* (Castellanos, 1960)	skin	53	[21]
*Sepiella inermis* (Van Hasselt, 1835)	skin	16.2*	[22]
Elasmobranchii			
*Chiloscyllium punctatum* (Müller and Henle, 1838)	cartilage	9.59*	[23]
*Carcharhinus limbatus* (Müller and Henle, 1839)	cartilage	10.3*	[23]
Echinoidea			
*Paracentrotus lividus* (Lamarck, 1816)	whole	7*	[24]
*Anthocidaris crassispina* (Agassiz, 1864)	whole	35*	[25]
Asteroidea			
*Patiria pectinifera* (Muller and Troschel, 1842)	body wall	6.1	[26]
Scyphomedusae			
*Aurelia aurita* (Linnaeus, 1758)	whole	0.01	[27]
*Chrysaora* sp.	bell	9–19	[28]
*Pelagia noctiluca* (Forsskål, 1775)	whole	0.07	[27]
*Catostylus tagi* (Haeckel, 1869)	bell	2.7*	[29]
	bell	4.5	[27]
*Cotylorhiza tuberculate* (Macri, 1778)	oral arms	19.4	[27]
	bell	< 10*	[27]
	bell	8.3–31.5	[27]
*Rhizostoma pulmo* (Macri, 1778)	oral arms	26–90	[27]
	bell	< 10*	[27]
*Rhopilema asamushi* (Uchida, 1927)	-	35.2*	[30]
*Rhopilema esculentum* (Kishinouye, 1891)	mesoglea	0.28*	[31]
*Stomolophus meleagris* (Agassiz, 1860)	mesoglea	46.4*	[32]
*Nemopilema nomurai* (Kishinouye, 1922)	mesoglea	2.2*	[33]

**Table 2:** Distribution of the most common collagen types within mammalian tissues.

Collagen Type	Tissue
I	Bone, skin, tendon, ligaments, cornea
II	Cartilage, vitreous body, nucleus pulposus
III	Skin, vessel walls, reticular fibers of lungs, liver, spleen
IV	Basement membranes
VI	Cornea (often associated with type I collagen)
